# HepaCAM inhibits cell proliferation and invasion in prostate cancer by suppressing nuclear translocation of the androgen receptor via its cytoplasmic domain

**DOI:** 10.3892/mmr.2022.12746

**Published:** 2022-05-23

**Authors:** Qingfu Deng, Li Luo, Zhen Quan, Nanjing Liu, Zhongbo Du, Wei Sun, Chunli Luo, Xiaohou Wu

Mol Med Rep 19: 2115–2124, 2019; DOI: 10.3892/mmr.2019.9841

Following the publication of the above article, an interested reader drew to the authors’ attention that the western blotting data shown in Figs. 2F and 5E were strikingly similar, even though they were intended to show the results from differently performed experiments; furthermore, the migration assay images shown for the 24 h ‘Blank’ and ‘Vector’ experiments in Fig. 3C were apparently the same.

The authors have consulted their original data, and realized that the errors in the presentation of these figures arose inadvertently as a consequence of selecting the wrong images for the 24 h ‘Blank’ experiement in Fig. 3C and the western blot for the AR / Nucleus experiment in Fig. 5E. The revised versions of Figs. 3 and 5 are shown on the next two pages. All the authors approve of the publication of this corrigendum, and the authors are grateful to the Editor of *Molecular Medicine Reports* for granting them the opportunity to publish this. The authors regret that these errors were included in the paper, and also apologize to the readership for any inconvenience caused.

## Figures and Tables

**Figure 3.**
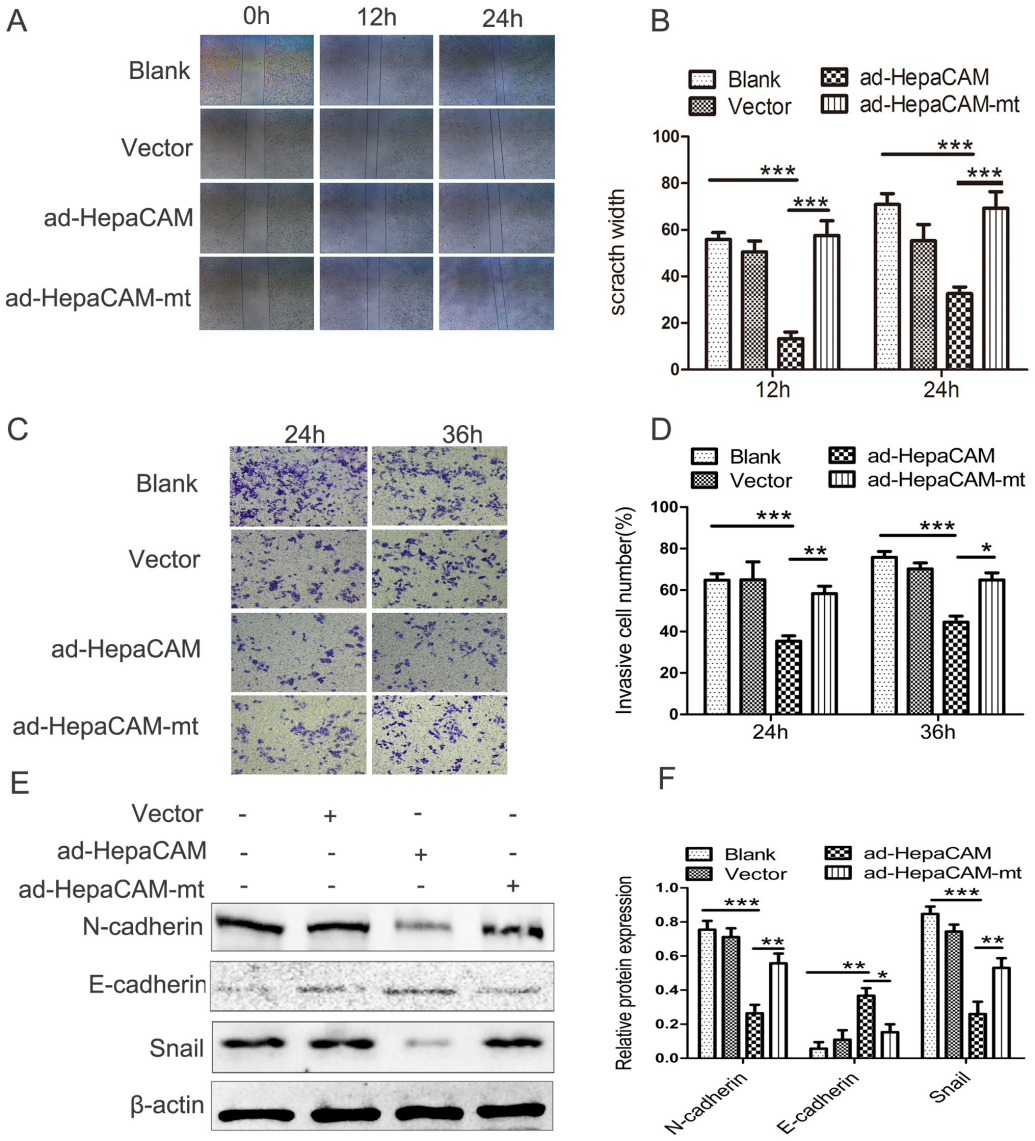
HepaCAM overexpression inhibits invasion and migration of LNCaP cells. (A and B) Wound healing assay of the LNCaP cells with different treatments. (C and D) Migrated LNCaP cells, as measured by Matrigel migration assay. (E and F) Expression levels of E-cadherin, N-cadherin, and Snail, as analysed by western blotting. The data are presented as the mean ± standard deviation. Magnification, ×200. *P<0.05, **P<0.01, ***P<0.001. Ad, adenovirus; HepaCAM, hepatocyte cell adhesion molecule; mt, mutant..

**Figure 5.**
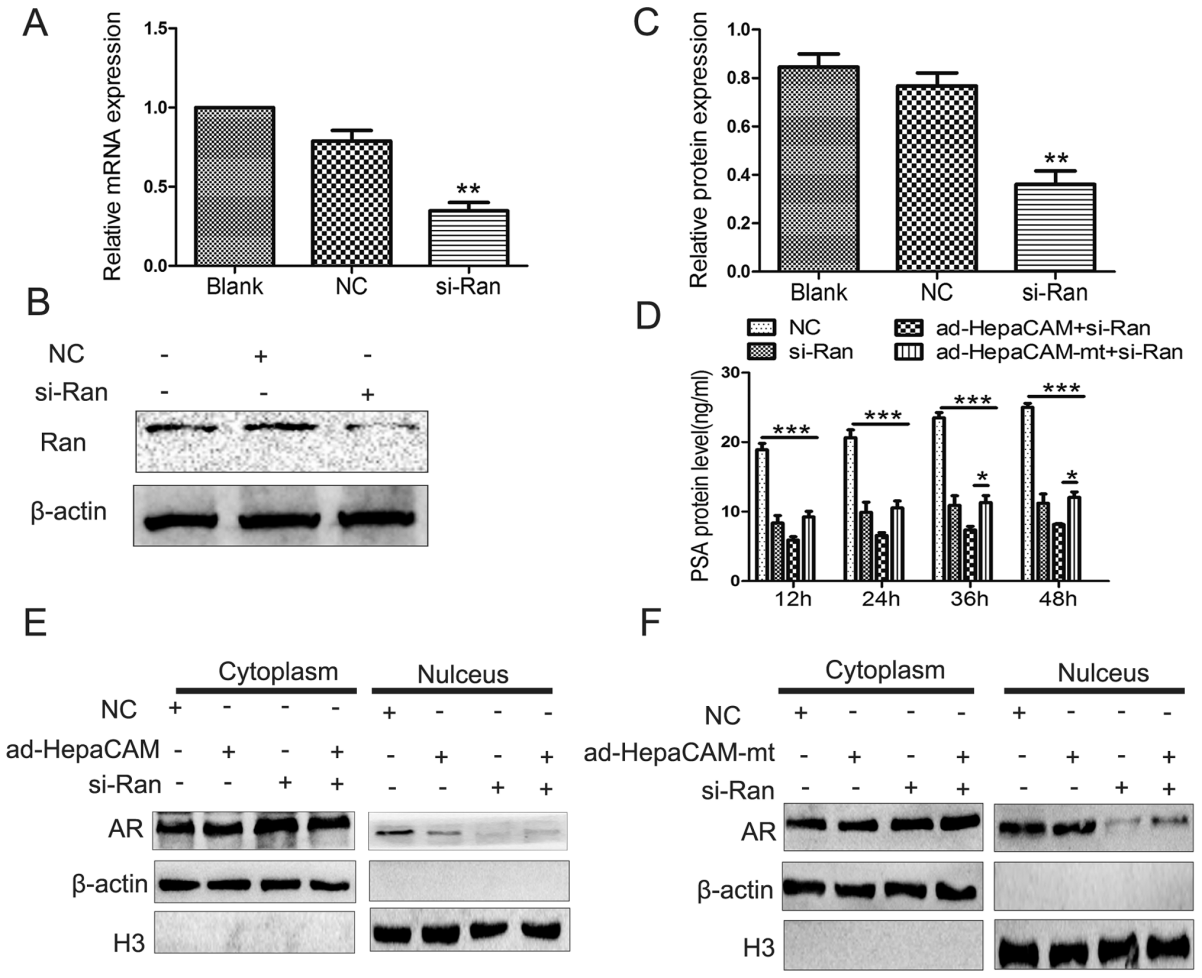
Differences in AR subcellular localization associated with si-Ran. (A) Relative mRNA expression levels of Ran was determined using reverse transcription-quantitative polymerase chain reaction after Ran was silenced by si-Ran in the LNCaP cells. (B and C) Ran protein expression was analysed using western blotting after Ran had been silenced by si-Ran in the LNCaP cells. (D) ELISA analysis of PSA was performed in the culture medium supernatant of the different groups at 12–48 h. Subcellular localization of AR protein in the LNCaP cells treated with si-Ran in the presence or absence of (E) ad-HepaCAM and (F) ad-HepaCAM-mt was analysed using western blotting. The data are presented as the mean ± standard deviation. *P<0.05, **P<0.01 and ***P<0.001. Ad, adenovirus; AR, androgen receptor; HepaCAM, hepatocyte cell adhesion molecule; mt, mutant; NC, negative control; PSA, prostate specific antigen; si, small interference.

